# The influence of BACE1 on macrophage recruitment and activity in the injured peripheral nerve

**DOI:** 10.1186/s12974-021-02121-2

**Published:** 2021-03-15

**Authors:** John A. Fissel, Mohamed H. Farah

**Affiliations:** Department of Neurology, Johns Hopkins University School of Medicine, The John G. Rangos Sr. Building, Room 239, 855 N. Wolfe Street, Baltimore, MD 21205 USA

**Keywords:** Peripheral nerve regeneration, BACE1, Sheddases, Macrophages

## Abstract

Following peripheral nerve injury, multiple cell types, including axons, Schwann cells, and macrophages, coordinate to promote nerve regeneration. However, this capacity for repair is limited, particularly in older populations, and current treatments are insufficient. A critical component of the regeneration response is the network of cell-to-cell signaling in the injured nerve microenvironment. Sheddases are expressed in the peripheral nerve and play a role in the regulation if this cell-to-cell signaling through cleavage of transmembrane proteins, enabling the regulation of multiple pathways through *cis*- and *trans*-cellular regulatory mechanisms. Enhanced axonal regeneration has been observed in mice with deletion of the sheddase beta-secretase (BACE1), a transmembrane aspartyl protease that has been studied in the context of Alzheimer’s disease. BACE1 knockout (KO) mice display enhanced macrophage recruitment and activity following nerve injury, although it is unclear whether this plays a role in driving the enhanced axonal regeneration. Further, it is unknown by what mechanism(s) BACE1 increases macrophage recruitment and activity. BACE1 has many substrates, several of which are known to have immunomodulatory activity. This review will discuss current knowledge of the role of BACE1 and other sheddases in peripheral nerve regeneration and outline known immunomodulatory BACE1 substrates and what potential roles they could play in peripheral nerve regeneration. Currently, the literature suggests that BACE1 and substrates that are expressed by neurons and Schwann cells are likely to be more important for this process than those expressed by macrophages. More broadly, BACE1 may play a role as an effector of immunomodulation beyond the peripheral nerve.

## Background

**Fig 1.**
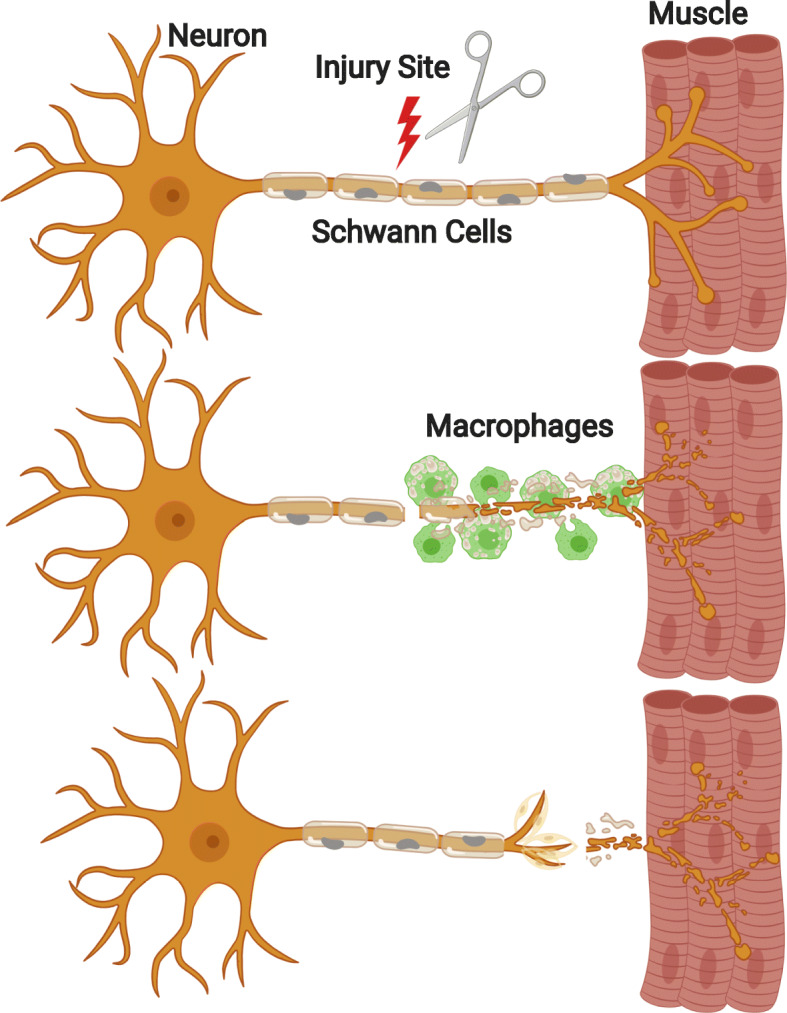
Distal nerve segment post-nerve injury. A motor neuron (left) innervates muscle tissue (right) via an axon myelinated by Schwann cells (center). After sustaining an injury, the distal nerve segment will degenerate into amorphous debris. Hematogenous macrophages (green) are recruited from circulation and enter the distal stump. Macrophages make the microenvironment more permissive for axonal regeneration by clearing cellular debris and releasing cytokines and neurotrophic factors. Axons regenerate with the guidance of Schwann cells and the aid of macrophages that release neurotrophic factors and eliminate debris that poses a physical barrier and releases inhibitory signaling to the regenerating axon

The peripheral nervous system (PNS) has a limited capacity for regeneration in response to injury that is insufficient to restore full function and mitigate morbidity and disability in humans. Following peripheral nerve injury, extensive coordination of several different cell types is required for the degeneration and subsequent regeneration of the peripheral nerve, beginning with the breakdown of axons and myelin sheaths into amorphous debris. Schwann cells differentiate into a pro-repair state and begin releasing cytokines and chemokines to recruit macrophages to the injured nerve. Next, in order to create a microenvironment conducive to axonal regeneration, recruited macrophages begin to clear cellular debris and release neurotrophic factors to promote axonal regeneration (Fig. 1). While neurons and Schwann cells play a critical role in the regeneration process and have been subject of recent review [1], the focus of this review will be on the role of macrophages in peripheral nerve regeneration, how they interact with neurons and Schwann cells, and role of sheddases, such as BACE1, in modulating these cell-to-cell interactions.

Beta-site amyloid precursor protein (APP) cleaving enzyme 1, also known as β-secretase (BACE1), has been extensively studied for its role in Alzheimer’s disease [2–5]. Cleavage of APP by BACE1 and subsequent cleavage by γ-secretase leads to the generation of Aβ peptides that form Aβ plaques that are a pathological hallmark of Alzheimer’s disease. In addition to this role in disease, BACE1 activity is involved in other biological processes and is expressed not only by neurons, but by other cell types such as Schwann cells [2, 6, 7]. The genetic deletion or pharmacological inhibition of BACE1 results in increased recruitment of macrophages and subsequent cellular debris clearance in the distal nerve segment [8]. While this phenotype has been shown to promote axonal regeneration [8], little is known about the underlying mechanism of this enhanced macrophage recruitment and debris clearance. The major cell types that comprise the microenvironment of the injured peripheral nerve and could be mediating this phenotype are neurons, Schwann cells, and hematogenous infiltrating macrophages; however, the relative contributions of each remain unknown. In order to elucidate a potential mechanism, it would be most prudent to start with possible signaling pathways that contain presently known BACE1 substrates that have immunomodulatory capacity.

## Events following peripheral nerve injury

### Degeneration following injury

Upon nerve injury, there is an initial lag period before the segment of nerve distal to the site of injury begins to degenerate [9]. The first step of this degeneration process is a flux of extracellular Ca^2+^ into the axon [10]. The depletion of extracellular calcium or blockage of Ca^2+^ ion channels can attenuate the degeneration of axons for up to 4 days [9]. The effectiveness of channel blockage at delaying degeneration illustrates that this degeneration process is due to the active function of Ca^2+^ channels as opposed to passive Ca^2+^ flux resulting from a compromised axonal membrane [9]. A consequence of this increased intracellular calcium is the activation of calpains, a family of proteases that are known to cleave cytoskeletal proteins such as neurofilament [9, 11–13].

Great advancement towards understanding the mechanisms of Wallerian degeneration have emerged from the discovery of the C57BL/*Wld*^*s*^ mouse, a spontaneous mouse mutant that exhibits very slow Wallerian degeneration [14]. Wallerian degeneration refers to the breakdown and granular disintegration of the distal nerve stump following axonal injury [15, 16]. *Wld*^*s*^ is a fusion protein that has the enzymatic activity of nicotinamide mononucleotide adenylyltransferase (NMNAT) and a modified N-terminal sequence that results in the relocation of the protein from its normal physiological location in the cell body to the cytoplasm of axons [17–19]. The cytoplasmic isoform of NMNAT, NMNAT2, is actively transported from the cell body to axons and catalyzes the formation of nicotinamide adenine dinucleotide (NAD+) from nicotinamide mononucleotide (NMN) [20, 21]. Once an axon is severed, this transport is blocked, and due to its short half-life, NMNAT2 rapidly degrades, resulting in decreasing NAD+ levels and rising NMN levels. This leads to axonal disintegration that can be prevented by the addition of exogenous NAD+ [22]. It has been proposed that *Wld*^*s*^ is able to protect axons due to its axonal localization and increased stability compared to NMNAT2, allowing the continued synthesis of NAD+ and delaying Wallerian degeneration [20, 23]. Interestingly, there is evidence that degeneration can be promoted by the accumulation NMN as opposed to depletion of NAD+, as illustrated by the axonal protection provided when NMN-synthesizing enzyme inhibitors are used, despite the downstream reduction in NAD+ [21].

Further advancement came from the discovery that blockage of Wallerian degeneration could be achieved through the deletion of Toll-like receptor adaptor protein Sterile alpha and TIR motif containing 1 (SARM1) [24]. This provided evidence of a mechanism of Wallerian degeneration that was driven by normal biological processes rather than occurring as the result of a mutated fusion protein. SARM1 deletion can also rescue the axonal degeneration resulting from loss of NMNAT2 without reducing NMN [25], suggesting SARM1 plays a role in previously known pathways discovered in *Wld*^*s*^ mice [26]. SARM1 and NMN appear to be required for generating a flux of extracellular Ca^2+^ into the axon in response to injury, which could drive calcium dependent effectors suggested by [9]. Further, SARM1 has been shown to have NADase activity which, following injury, results in the depletion of NAD+ and subsequent axonal degeneration [27].

The process of axonal degeneration likely plays a role in the modulation of Schwann cells following injury through an injury signal or loss of axonal contact signal [1, 28]. In response to injury, Schwann cells will rapidly switch their transcriptional program, differentiate to a repair state, and produce cytokines for the mobilization of immune system cells for regeneration [29–32]. Schwann cells are also one of the earliest mediators of debris clearance before hematogenous macrophages arrive in the distal nerve segment. Repair-state Schwann cells further promote the later stages of regeneration by elongating and forming a guidance tube for the regenerating axon. Following successful reinnervation of the target by the axon, Schwann cells will revert to a myelinating state in response to signaling from the maturing axon.

There has been recent interest in the role of sheddases, such as BACE1, members of the A disintegrin and metalloproteinase (ADAM) family, and gamma secretases in the process of nerve regeneration. This topic has been comprehensively detailed in the following review [33]. Sheddases are known to be regulators of Schwann cells, particularly in the context of myelination. During development, BACE1 cleavage of NRG-1 has been shown to promote myelination [6, 34], while cleavage by another sheddase, ADAM 17, inhibits myelination [35, 36]. Sheddases have also been shown to play a role following peripheral nerve regeneration. It has been demonstrated that ADAM 19 activity is involved in the signaling process that aids in the differentiation of Schwann cells back to a myelinating state [37]. Sheddase activity may also impact axonal physiology, as ADAM 10 has been shown to promote axonal sprouting and outgrowth in vitro [38]. While the impact of sheddases on cell signaling in peripheral nerve regeneration is a growing topic, this review will focus on how BACE1 may modulate the behavior and activity of macrophages.

### Recruitment and activity of macrophages following nerve injury

Following nerve injury macrophages begin to infiltrate the distal segment after 2–3 days, peak around 6 days, and persist in large numbers for at least 14 days [39]. To date, several cytokines and chemokines have been demonstrated to have a role in macrophage recruitment to the injured peripheral nerve, including tumor necrosis factor α (TNFα), interleukin-1α (Il-1α), interleukin-1β (Il-1β), C-C motif chemokine ligand 2 (CCL2), leukemia inhibitory factor (LIF), and pancreatitis-associated protein III (PAP-III) [29–32, 40–42]. In experiments where these cytokines are blocked, macrophage recruitment is attenuated to varying degrees, but the loss of any single molecule does not completely block recruitment [30]. While the reduction of any cytokine or chemokine individually will not completely abrogate the recruitment of macrophages, it does illustrate that this is a robust response that is the result of macrophage mobilization through multiple pathways [29].

Macrophages are vital to the process of regeneration because when recruitment is attenuated, there is reduced myelin debris clearance, axonal regeneration and functional recovery [30, 43]. The removal of myelin and axonal debris in the distal nerve segment is a critical component of peripheral nerve regeneration. Not only is this debris a physical barrier to the regenerating nerve, but the debris also contains inhibitory signaling molecules that further obstruct the regenerating axon [44–47]. The bulk of this process takes place within 20 days following injury in mouse models [30, 39, 48]. Debris clearance largely occurs in two phases: the first driven by Schwann cells and resident macrophages in the first few days and the second driven by infiltrating hematogenous macrophages in the following days [39, 49–54]. Upon arrival into the distal nerve stump, macrophages begin phagocytosis of axonal and myelin debris. This process is, in part, driven by traditional opsonizing elements of the humoral immune system, namely antibodies [39] and complement [55, 56]. Beyond opsonin-dependent phagocytosis of myelin debris, there is also evidence of the involvement of the versatile scavenger receptor in the mediation of phagocytosis [57, 58]. It is not clear if any other opsonin-independent pathways are involved in the phagocytosis of cellular debris by macrophages, but Tyro3, Axl, Mer (TAM) receptor-mediated phagocytosis has been observed in Schwann cells, so similar mechanisms may be relevant to macrophages [48].

Macrophages also contribute neurotrophic signaling to the regenerating nerve environment in a variety of ways. Conditioned media produced by macrophages that have phagocytosed myelin debris can increase survival and neurite quantity of dorsal root ganglia neurons in culture [59]. Macrophages also play a critical role in sensing the hypoxic environment caused by the disruption of blood vessels. Macrophages produce vascular endothelial growth factor (VEGF) in response to hypoxia, stimulating polarized angiogenesis of blood vessels that form a scaffold for Schwann cells to follow in order to reach the distal nerve segment following nerve transection [60]. Another cohort of macrophages will persist in the distal nerve segment for at least 6 weeks following injury and provide pro-regenerative signaling molecules to the regenerating axons and Schwann cells [60, 61]. These persisting macrophages aid in regulating the return of Schwann cells to a myelinating differentiation state to myelinate regenerating axons. Lastly, macrophages are involved in a heavily regulated remodeling process of the extracellular matrix (ECM). This process involves a delicate balance of enzymes involved in digestion of ECM components, known as matrix metalloproteinases (MMPs), and their inhibitory counterparts [62]. The degenerating-nerve basement membrane needs to be permeable enough to allow the influx of hematogenous macrophages but must remain intact to provide a niche in which the Schwann cells can proliferate and await the time to remyelinate regenerating axon [62, 63].

### β-secretase

BACE1 is a well-studied enzyme in the field of Alzheimer’s disease. However, this enzyme’s promiscuous substrate specificity results in potential roles in multiple processes beyond the pathology of Alzheimer’s disease. This enzyme is a transmembrane aspartyl protease that is a critical participant in the cleavage of APP and generation of Aβ plaques, one of the pathological hallmarks of Alzheimer’s disease. Initially, BACE1 cleaves APP on the luminal side of the cell membrane, releasing a large soluble ectodomain and leaving behind the remaining truncated transmembrane and intracellular domains. This cleavage event is the rate limiting step in the generation of Aβ fragments. Subsequent cleavage of the transmembrane domain by gamma secretase will generate the Aβ fragment and APP intracellular domain [64]. BACE1 substrates are mostly type 1 transmembrane proteins; however, there are exceptions like St6gal-1, which is a type 2 transmembrane protein [65]. Its broad substrate specificity has led to the discovery of over 60 known substrates of BACE1 [66], and this number continues to grow [64, 67, 68]. BACE1 has been found to have the highest expression in brain and the pancreas, although it has high and low enzymatic activity in each, respectively [69]. Expression is present in other peripheral tissues but only very low amounts. Genetic and pharmacological manipulation of BACE1 has revealed several phenotypes that are of particular interest to the process of peripheral nerve regeneration. Investigation of these phenotypes thus far has centered on the same principal components of nerve regeneration: intrinsic neuronal regeneration capacity, inhibitory debris clearance, and pro-regeneration effects of Schwann cells.

The role of BACE1 in peripheral nerve regeneration has been studied in mouse BACE1 KO models, including a global BACE1 KO and a macrophage-specific BACE1 KO model (Table 1). BACE1 KO neurons have a greater capacity for regeneration than their wild type counterparts. Following a crush injury, BACE1 KO axons grow significantly further distally than WT and this difference increases over longer time points [8]. Not only do the axons grow more rapidly, but there is also a greater number of regenerating axons in the BACE1 KO, resulting in greater reinnervation of neuromuscular junctions (NMJs) [8]. Similarly, pharmacological inhibition of BACE1 in mice following nerve injury results in increased numbers of regenerating axons, reinnervated NMJs, and improved functional recovery [71]. BACE1 inhibition also enhances axonal sprouting, a process by which intact axons send out sprouts that reinnervate nearby NMJs [71, 72]. While reduced BACE1 activity has a pro-regenerative effect, the opposite is true of BACE1 overexpression. Overexpression of human BACE1 in neurons of transgenic mice results in decreased axonal regeneration, reduced NMJ reinnervation, and impaired functional recovery following crush injury [73].

**Table 1:** Phenotypes of global BACE1 KO vs. macrophage specific BACE1 KO mice

Global BACE1 KO	Macrophage specific BACE1 KO
**Hypomyelination at baseline** **[**6, 34]	**Normal myelination** [70]
**Enhanced axonal regeneration** [8]	**Not determined**
**Reduced remyelination** [7]	**Not determined**
**Enhanced macrophage recruitment** [8]	**No enhanced macrophage recruitment** [70]

In addition to its role in axons, BACE1 is also expressed by Schwann cells and plays a role in their regulation. In BACE1 KO mice, peripheral nerves are hypomyelinated due to dysregulation of neuregulin 1 type III [6, 7], a known BACE1 substrate that plays a role in the myelination of axons [74, 75]. BACE1 KO mice also show increased proliferation of Schwann cells during development compared to WT [76].

In BACE1 KO mice, there is an increase in macrophages recruited to the distal nerve segment following nerve injury, although these macrophages do not appear to arrive any earlier than expected. In vivo morphological data suggests that BACE1 KO macrophages are more phagocytically active, as evidenced by an increase in the number of macrophages with a myelin-laden foamy appearance. This increased phagocytosis is also observed in vitro in primary mouse intraperitoneal macrophages [8]. However, it is unclear if these macrophages are able to clear cellular debris more efficiently than wild type (WT) counterparts because BACE1 KO mouse axons are hypomyelinated, making direct comparison more challenging [6, 34]. The improved clearance of cellular debris may be the result of the hypomyelination phenotype, as there is less debris to be cleared by macrophages overall. At this time, it is unclear of whether BACE1 activity modulates macrophages directly or indirectly.

In order to determine the mechanisms of this phenotype, a critical first step is to determine what the consequences are of reduced BACE1 activity in the various cell types of the regenerating nerve microenvironment. A nerve grafting experiment was done where BACE1 KO or WT sciatic nerves were grafted into a recipient sciatic nerve. Interestingly, there was increased axonal growth when a WT graft was placed into a BACE1 KO host but not vice versa [8]. This suggests that the BACE1 activity status of the recipient is more important than that of the donor. The likely cell types mediating this recipient effect are the neurons and/or macrophages. Neuronal cell bodies are located proximal to the graft tissue and macrophages are recruited from recipient circulation. This experiment suggests that BACE1 activity does not have a strong effect on Schwann cells in enhancing axonal regeneration [8]. However, macrophage-specific deletion of BACE1 is not sufficient to recapitulate the enhanced macrophage recruitment phenotype observed in global BACE1 KO mice [70], suggesting that macrophages are not driving this phenotype. To date, a clear picture of which cell types and potential mechanisms are driving the enhanced peripheral nerve regeneration phenotype of BACE1 KO mice has not yet emerged.

## BACE1 substrates with immunomodulatory potential

### Interleukin 1 receptor 2 (Il1-R2)

The interleukin 1 (Il-1) signaling pathway and its decoy receptor Il1-R2 have been extensively studied in the context of the immune system and inflammation [77, 78]. This pathway has also been the subject of thorough review that discusses it in much greater detail than will be done here [77]. Il1-R2 is a substrate of BACE1 [79], and its cleavage could influence many parts of this signaling pathway. Not only could cleavage of Il1-R2 by BACE1 alter *cis*-Il-1 signaling in cells expressing Il-1R2, but it could also influence *trans*-Il-1 signaling in the injured peripheral nerve microenvironment by releasing shed Il1-R2 (shIl1-R2) into the extracellular milieu after cleavage.

**Fig. 2:**
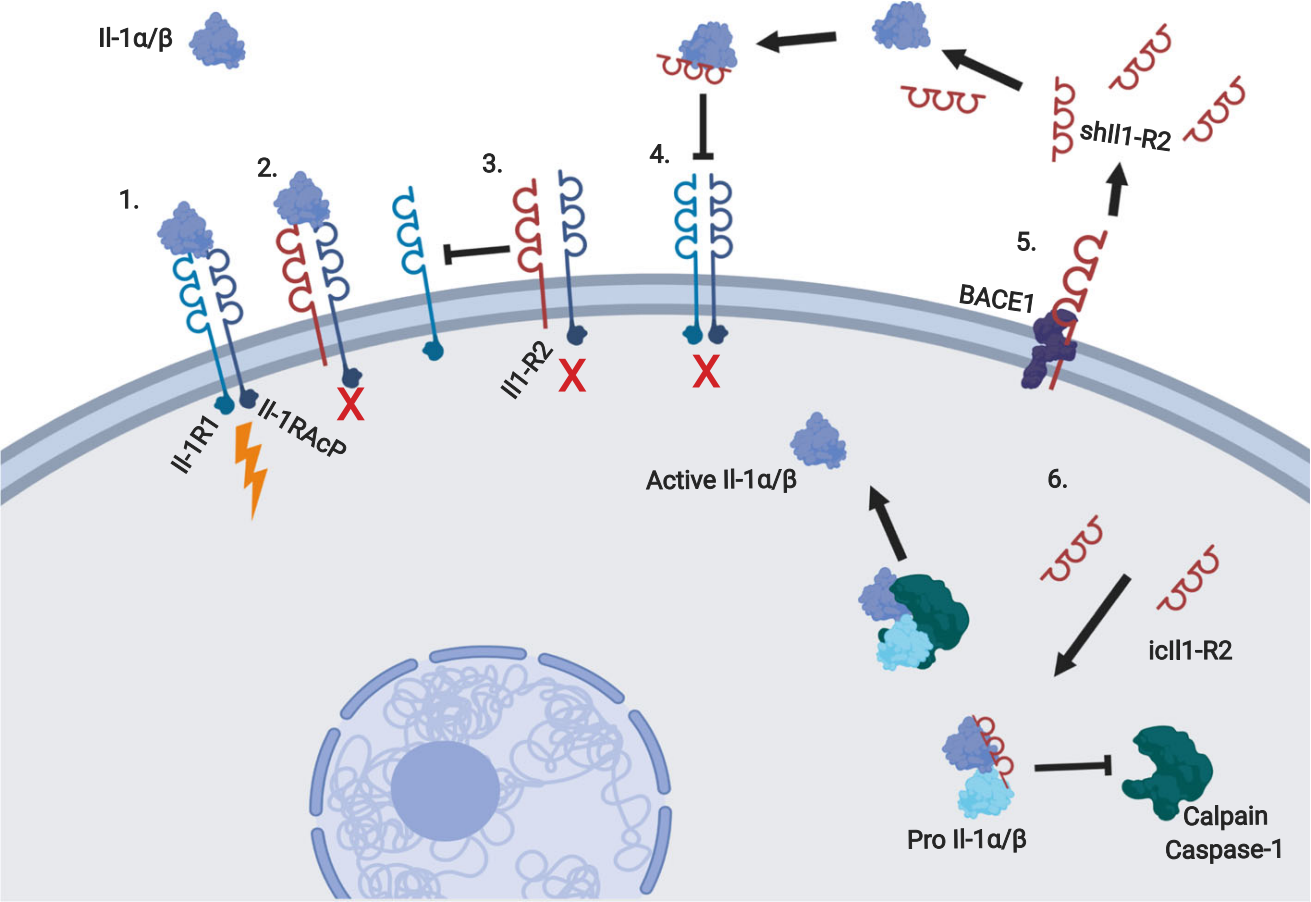
Postulated regulatory mechanisms of Il-1 by BACE1 activity. Il1-R2 regulation of Il-1 signaling. (1) Il-1α or Il-1β binds Il1-R1 and Il-1RAcP, resulting in normal signaling. (2) Il1-R2 binds Il-1RAcp and either Il-1α or Il-1β but does not result in signaling. (3) Il1-R2 can regulate Il-1 signaling by binding Il-1RAcP and which leaves it unable to complex with interleukin 1 receptor (Il-1R1) for signaling. (4) Il1-R2 can be secreted or shed by cells and can bind Il-1α and Il-1β extracellularly and prevent them from binding Il-1R1/Il-1RAcP. (5) BACE1 is one of a few enzymes capable of cleaving Il1-R2. BACE1 activity could influence the relative amounts of Il-R2 present in the cell membrane or shed into extracellular space. (6) Intracellular Il1-R2 can bind either pro-Il-1α or pro-Il-1β and prevent cleavage by calpain or caspase-1, respectively, for activation

Membrane bound Il1-R2 can be cleaved into shIl1-R2 by different “sheddases,” such as ADAM17 or BACE1 [65, 79, 80]. This makes sheddases very important components of this regulatory system as they not only serve to restore *cis*-Il-1 signaling at the cell membrane, but they also may be important for generating shIl1-R2 which can bind extracellular Il-1α and Il-β and attenuate *trans*-Il-1 signaling. This suggests the possibility of a context dependent pleotropic role for sheddases, like BACE1, in the regulation of the Il-1 signaling pathway, which could have implications for the immunomodulatory phenotype observed in the injured peripheral nerve of BACE1 KO mice (Fig. 2).

Il-1 plays an important role in peripheral nerve regeneration. Following peripheral nerve injury, there is rapid and robust induction of Il-1β expression in a small population of cells near the injury site [30]. This response occurs after 1 h, which is nearly 12 h prior to arrival of neutrophils and later hematogenous macrophages. Given the timing of this expression change, it is unlikely to be hematogenous leukocytes, although there is a second peak of Il-1β expression at 14 days that is likely due to influx of hematogenous macrophages [30]. It is possible that these Il-1β generating cells could be a subset of Schwann cells or fibroblasts, as expression of Il-1β has also been observed in Schwann cells following injury [81]. However, macrophages are known to express Il-1β in response to inflammatory stimuli such as Il-1α or other endogenous molecules released into extracellular space, and resident macrophages account for approximately 10% of all nucleated cells in uninjured nerves [52, 54, 82], so the sparsity of cells that are heavily expressing Il-1β would make these cells a likely source [81]. Il-1α is constitutively expressed in the nerve so it would be present to serve as a damage-associated molecular pattern (DAMP) to prime Il-1β expression following injury. Mature Il-1β levels begin to increase after 6 h and continue to rise for 24 h [31, 81]. This maturation mirrors the processing of pro-caspase, which suggests that this increased expression of Il-1β is mediated by activation of the inflammasome. Mice that have defects in Il-1 signaling have impaired functional recovery following nerve injury compared to WT, highlighting the benefit to this inflammatory signaling [31]. On the other hand, Il-1β also plays a role in neuropathic pain as the result of inflammation. While Il-1β/TNFα double KO mice display evidence of impaired functional recovery, they also have reduced mechanical allodynia [31]. However, depletion of neutrophils, as opposed to blockage of cytokine signaling, improved neuropathic pain thresholds and restored functional recovery to WT levels [31].

This complex signaling pathway presents many opportunities for BACE1 to play a regulatory role in peripheral nerve regeneration. BACE1 activity could boost the *trans-*regulatory effect of Il1-R2 on adjacent cells through shedding of Il1-R2 into the extracellular milieu. The opposite could be true of its *cis*-regulatory activity. Reduced BACE1 expression could lead to more Il-R2 remaining on the cell surface to block that cell’s ability to respond to Il-1 and sinking interleukin 1 receptor accessory protein (Il-1RAcP). It is not fully understood how intracellular Il1-R2 (icIl1-R2) is generated, so perhaps BACE1 may play a role, given its ability to generate shIl1-R2. Increased levels of icIl1-R2 could interfere with the calpain-mediated maturation of Il-1α. This would reduce the ability of cells experiencing calcium flux to use Il-1α for DAMP signaling and would have downstream consequences for the inflammatory process as a whole. Decreasing BACE1 expression could reduce the amount of available icIl1-R2 and promote a more robust inflammatory response to injury.

It does not appear that eliminating BACE1 expression in macrophages plays a role in modulating the inflammatory response, as macrophage-specific deletion of BACE1 did not result in the same increased macrophage recruitment observed in global BACE1 KO mice [70]. This suggests that the *cis*-regulatory activity of Il1-R2 in macrophages would not be sufficient to alter recruitment behavior. Further, it is also possible that macrophages do not participate in *trans-*regulation of the peripheral nerve microenvironment through the shedding of shIl1-R2. The implications of this evidence shifts the focus of potential Il1-R2 regulation of the immune response towards the other cells of the injured nerve microenvironment, such as Schwann cells, and axons, which are both known to express BACE1.

The Il1-R2 signaling pathway is heavily regulated and has many redundancies that would limit the ability of BACE1 to have a dramatic impact on any single regulatory site, but perhaps BACE1 can influence the pathway at several different regulatory sites and drive the overall response in a pro-regenerative direction by leveraging the benefits of the inflammatory response, while minimizing its detrimental effects. Optimal regeneration seems to be heavily reliant on very tight regulation of the inflammatory response following injury. If there is not enough of an inflammatory response, then there is attenuated immune cell recruitment for trophic signaling and debris clearance. However, if the inflammation response is too great, then there can be deleterious effects such as the development of neuropathic pain [31]. Any alterations of the immune response for therapies will require a breadth of knowledge on how the various cell types and pathways are interconnected and influence one another to maximize the balance of repair and pain mitigation.

### Jagged1 (Jag1)

There is increasing evidence of the importance of Notch signaling through its ligand Jag1 in the context of the peripheral nerve disorders. Mutations in Jag1 have been associated with peripheral neuropathy in humans [83]. Transgenic mice bearing the same mutations in Jag1 as affected patients display abnormal focally folded myelin [83]. More broadly, Notch signaling has been shown to play a role in the regulation of Schwann cells and post-natal myelination [84]. BACE1 cleavage of the Notch ligand, Jag1, results in significant changes in the behavior of Schwann cells [85, 86], which suggests BACE1 could potentially modulate macrophage-Schwann cell interactions. However, to date, there has not been a direct assessment of the role of Notch signaling in the recruitment of macrophages to the injured peripheral nerve. The following section will discuss this potential role.

Notch is a transmembrane protein that can be activated by the binding of a ligand on an adjacent cell. This binding results in a conformational change in Notch that makes it more vulnerable to a series of proteolytic cleavages that release the Notch intracellular domain (NICD) into the cytoplasm. The NICD subsequently relocates to the nucleus and activates target genes for transcription with the help of other coactivating proteins [87]. This pathway does not have a mechanism for amplification, so the strength of the signal is the result of the abundance of Notch receptors on the receiving cell and the amount of ligand on the sending cell. The ratio of Notch to ligand can also be an important part of the pathway’s regulation. *Cis*-inhibition of Notch occurs when a cell is expressing sufficient amounts of the ligand on its surface, and Notch binds to the ligand on the cell’s own surface rather than the ligand from an adjacent cell [87, 88]. As a result of *cis*-inhibition, the sending/receiving state of the cell can be largely determined by this receptor to ligand ratio. For example, if the amount of Notch receptor is greater than the amount of ligand, then that cell is likely to be a receiver; the opposite is true of cells with more ligand. Ligand binding efficiency provides another layer of regulation to the Notch pathway [89]. For example, Jag1 has a relatively low binding affinity for Notch, yet can render a cell unresponsive to other Notch ligands with greater binding affinities [90].

**Fig. 3:**
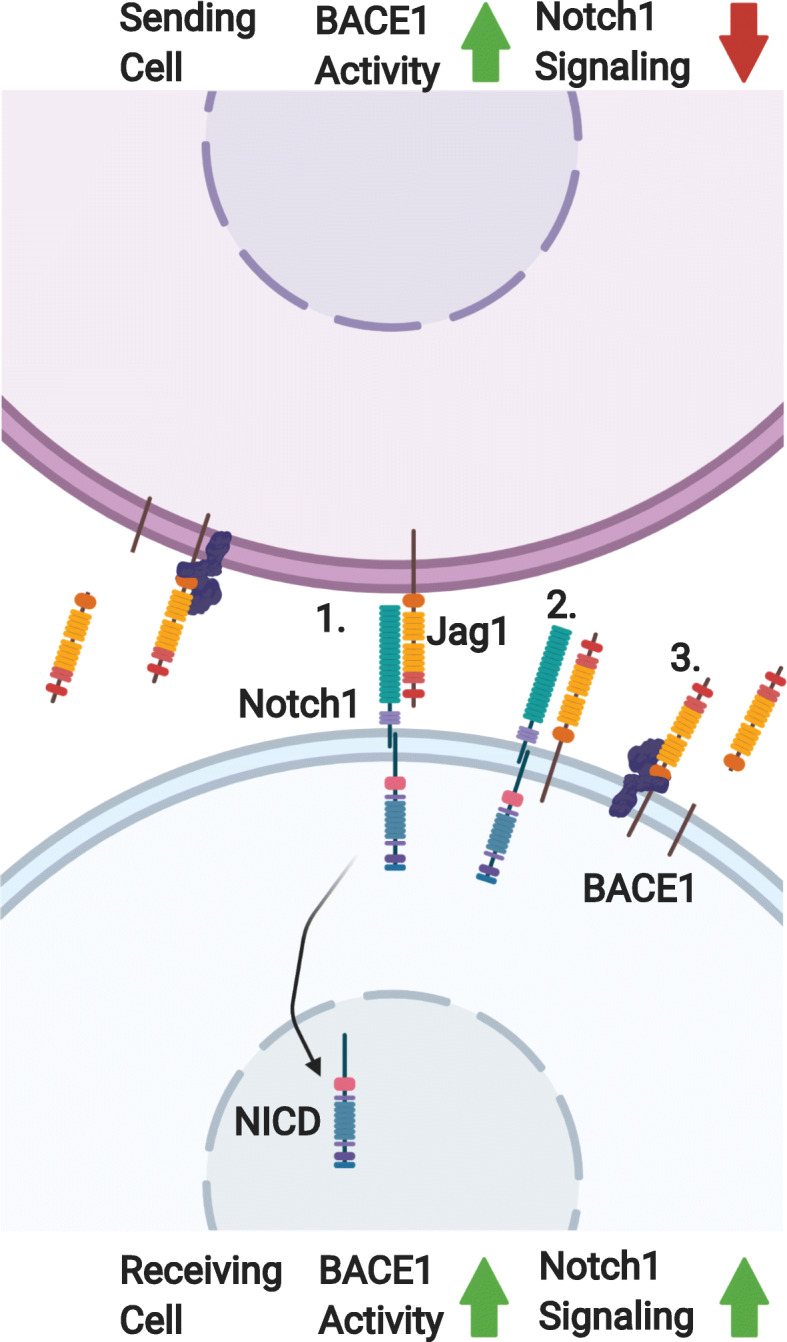
Jagged-1/Notch signaling and regulation in the context of BACE1 activity. A cell that is sending (top) a Notch signal to a receiving cell (Bottom) via notch ligand Jag1. (1) Notch/Jag1 binding causes a conformational change in Notch1 which results in subsequent cleavages, resulting in the release of the intracellular domain of Notch (NCID) to relocate to the nucleus and activate downstream signaling. (2) Notch signaling can be regulated by binding Jag1 on the surface of the receiving cell. (3) BACE1 can regulate Notch1 signaling via the cleavage of Jag1. This cleavage can have different effects depending on whether it is taking place on a receiving cell or sending cell. In the sending cell, BACE1 activity results in reduced Jag1 available for signaling. In the receiving cell BACE1 activity results in increased Notch1 signaling by reducing the amount of *cis*-regulating Jag1

BACE1 has also been shown to play role in regulating the Notch signaling pathway via cleavage and shedding of Jag1 (Fig. 3) [86]. In BACE1 KO mice, there is an abundance of full-length Jag1 in the hippocampus. This abundance of the Jag1 ligand results in more Notch activation that is detected by increased NCID in this region of the brain. In vitro overexpression of BACE1 causes a decrease in the amount of full length Jag1, and this effect is reversed when cells are treated with a BACE inhibitor. BACE1 demonstrates specificity for Jag1, as it is unable to efficiently cleave its homolog Jag2. These studies suggest that BACE1 is a regulator of Notch signaling via cleavage of its substrate Jag1 [85, 86]. The increased Jag1 in BACE1 KO mice causes a shift in the ratio of Notch to ligand, resulting in a shift towards astrogenesis and away from neurogenesis in the developing brain. The ability for BACE1 to modulate glial cells in the central nervous system (CNS) bodes well for a similar role in the PNS. Beyond the regulation of CNS cell differentiation, there is also evidence of BACE1 regulation of Notch signaling playing a role in the PNS. As mentioned previously, BACE1 KO mice have hypomyelinated axons due to the loss of BACE1 dependent neuregulin 1 signaling. Notch has been shown to enhance the sensitivity of Schwann cells to NRG-1 [84]. In addition, Schwann cells show increased proliferation in BACE1 KO mice [76], which could be the result of increased Notch signaling due to the loss of BACE1 regulation of Jag1. BACE1 KO mice have increased full length Jag1 and Delta1, another Notch ligand, and downstream NICD in sciatic nerves compared to WT. The addition of Delta1 as a possible BACE1 substrate expands the impact that BACE1 can have on the Notch pathway [76].

Notch also influences the phagocytic activity of macrophages by reducing the expression of signal regulatory protein α (SIRPα). SIRPα is expressed by macrophages, dendritic cells, and some neurons and binds CD47, which is present in myelin debris, to block phagocytosis. This serves as a “don’t eat me” signal from the myelin debris. In SIRPα KO mice there is increased Wallerian degeneration and clearance of myelin debris following nerve injury [91]. The reduction of SIRPα results in a loss of the “don’t eat me” signal and increases the phagocytic activity of macrophages. SIRPα activity can also attenuate the ability of macrophages to respond to inflammatory stimuli involved in polarization of macrophages toward an inflammatory phenotype. This suggests that SIRPα activity is not only important for phagocytosis but also for more broad mediation of the inflammatory response [92].

Regulation of macrophages by Notch following peripheral nerve injury is still speculative at this point, but there are multiple ways it could occur. Macrophage-specific deletion of BACE1 does not result in increased macrophage recruitment [70], which suggests that *cis*-inhibition of Notch signaling by Jag1 in macrophages does not play a large role in mediating this enhanced recruitment. Schwann cells may play a role in the enhanced recruitment in global BACE1 KO mice, as they would have an abundance of Jag1 to signal macrophage-derived Notch. Further, this signaling between Schwann cells and macrophages could result in decreased expression of SIRPα that may explain the enhanced debris clearance observed in global BACE1 KO mice. Given the abundance of Notch receptors and Notch ligands, it is unlikely that BACE1 would have absolute regulatory control over any of these effects, but rather it could be a crucial mediator that helps tip the balance of signaling towards a pro-regenerative phenotype in the peripheral nerve. The link between mutations in Jag1 and peripheral neuropathies [83] also highlights an interesting avenue of clinically significant research to pursue in the future.

### ST6 beta-galactoside alpha-2,6-sialyltransferase 1 (ST6gal-1)

Cleavage of the BACE1 substrate, ST6gal-1 [93, 94], may provide evidence of a beneficial role of BACE1 activity following peripheral nerve injury. In inflammatory contexts, there is evidence that BACE1 activity enhances adhesion of monocytes to endothelial cell adhesion molecules and reduces endothelial cell-cell adhesion [95, 96]. ST6gal-1 is a sialyltransferase enzyme that plays a role in the post-translational modification of proteins and modification of glycolipids by catalyzing α2,6 sialylation of N-glycans [97–99]. While this enzyme is expressed in detectable amounts in nearly every tissue at a basal level, liver cells can rapidly upregulate expression of ST6gal-1 during the inflammatory acute phase response [97]. In addition to the increased expression driven by the inflammatory response, ST6gal-1 can also be cleaved and released into the serum [97]. While this cleavage is likely a form of regulation, there is also evidence that certain cleaved forms of ST6gal-1 retain enzymatic activity. A study of the sialylation of IgG in B cells [100] showed that when B cell ST6gal-1 was selectively ablated, there was still ST6gal-1 dependent sialylation of IgG in serum [98]. characterized an E41 cleaved form of secreted ST6gal-1 that was able to bind to an affinity column used to purify sialyltransferases, suggesting that this form retains its activity.

BACE1 has been shown to cleave ST6gal-1 in vitro, and in a study of BACE1 KO mice, there was a decrease in serum ST6gal-1 compared to WT. The opposite effect was observed in mice that overexpress human BACE1 [98]. BACE1 expression and serum ST6gal-1 levels were also shown to be increased in a rat model of hepatitis, supporting earlier studies regarding hepatic inflammation playing a role in ST6gal-1 expression [97]. BACE1 cleavage of ST6gal-1 results in the generation of an enzymatically active form of ST6gal-1, E41 [98].

**Fig. 4:**
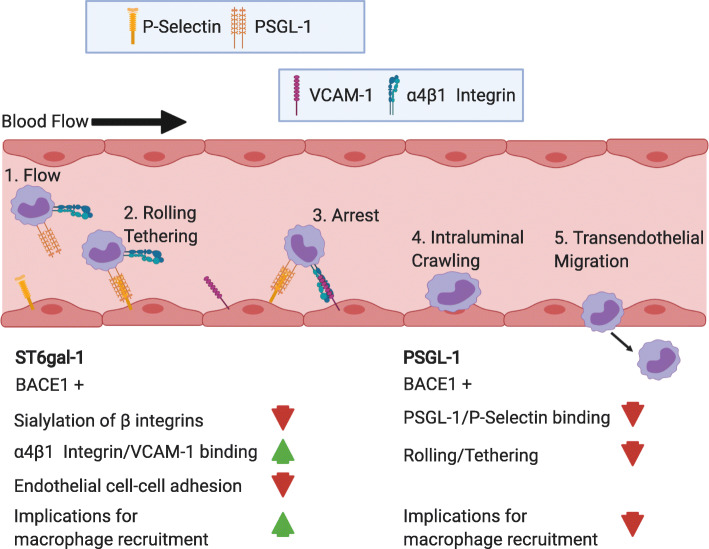
Potential implications of BACE1 cleavage of ST6gal-1 and PSGL-1 on macrophage recruitment. The binding cascade leading to transendothelial migration and recruitment of leukocytes, in this case monocytes, begins with binding interactions between PSGL-1 and P-selectin. These adhesion molecules disrupt flow of monocytes through the blood stream (1) by initiating the rolling and tethering to endothelial cells (2). This binding progresses until the arrest of the monocyte (3) with the addition of VCAM-1 and integrin binding. At this point, VCAM-1/integrin binding takes over and plays a critical role in facilitating transendothelial migration (4,5). BACE1 cleaves ST6gal-1, resulting in decreased sialyation of α4β1 integrin, which increases its binding affinity to VCAM-1. Further, this hyposialyation leads to decreased endothelial cell-cell adhesion. This combination of these effects would likely provide conditions more permissive to macrophage recruitment. Conversely, BACE1 cleavage of PSGL-1 would reduce the amount PSGL-1 available on the cell surface to bind with P-selectin. This would likely reduce the frequency of the initial binding reactions of this cascade and would likely attenuate macrophage recruitment

BACE1’s apparent role in the regulation of sialylation through its cleavage of ST6gal-1 can have a range of immunomodulatory outcomes relevant to the differentiation and recruitment of macrophages to the site of injury [96, 101]. The current literature suggests that with regard to ST6gal-1, BACE1 activity is beneficial for monocyte recruitment by enhancing macrophage/endothelial cell adhesion and promoting extravasation by decreasing endothelial cell-cell adhesion (Fig. 4) [96, 101]. In a study of the monocyte cell line U937, cells showed increased binding to VCAM-1 coated plates in a monocyte α4β1 integrin dependent manner following differentiation into a mature, activated state by phorbol myristate acetate (PMA). This binding interaction of α4β1 on monocytes to endothelial VCAM-1 is important for the trafficking of monocytes to tissues following an inflammatory response. Sialylation of β1 integrins has been shown to be key regulator of the adhesion reaction between the α4β1 molecules and VCAM-1 and fibronectin. Hyposialylated α4β1 shows increased binding affinity for VCAM-1. Upon differentiation of monocytes by PMA, there is an increased expression of BACE1 associated with macrophage activation via protein kinase C (PKC) pathway. This increased expression of BACE1 leads to increased cleavage of ST6gal-1 and hyposialylation of α4β1. This hyposialylation and subsequent increased binding affinity correlates temporally with the activation of PKC and can be reversed when cells are treated with a BACE inhibitor [96]. A different study by [101] highlighted the connection between the inflammatory response, BACE1 expression, and the modulation of cell adhesion. Treating a human monocyte cell line, THP-1 cells, with exogenous TNFα resulted in increased cell adhesion with endothelial cells in co-culture experiments. TNFα also caused a reduction in tight junction stability, observed morphologically via electron microscopy, and reduced immunofluorescent VE-Cadherin staining. BACE1 expression increased in response to TNFα which in turn cause a global reduction in 2,6 sialylation levels as observed by lectin staining and flow cytometry. This reduction in sialylation compromised endothelial cell-cell adhesion and enhanced monocyte adhesion to endothelial cells [101].

Evidence for the upregulation of BACE1 expression by the inflammatory response is building [70, 96, 101]. While macrophage trafficking seems to be enhanced by BACE1 cleavage and downregulation ST6gal-1, another component of monocyte trafficking, discussed in the next section, may be enhanced by deletion of BACE1. Although, it appears that ST6gal-1 cleavage by macrophages may not sufficient to change levels of recruitment from WT levels, as macrophage-specific deletion of BACE1 did not result in increased macrophage recruitment following nerve injury [70]. A clearer picture of potential benefits of BACE1 activity in the context of peripheral nerve injury has yet to emerge.

### P-selectin glycoprotein ligand-1 (PSGL-1)

P-selectin is a critical component for the trafficking of leukocytes out of the bloodstream and into tissues [102]. It is constitutively expressed by endothelial cells and its binding partner PSGL-1 is expressed by leukocytes, including monocytes. Transient binding interactions between p-selectin and its ligand PSGL-1 are critical for the initial rolling and tethering of monocytes to endothelial cells during recruitment to the injured nerve [103]. This suggests that altering the cleavage of, PSGL-1, a BACE1 substrate [102], could potentially influence the recruitment of macrophages to the injured peripheral nerve. While these binding interactions between p-selectin and PSGL-1 are short in duration, they result in the reduced velocities of leukocytes in the blood stream. This reduced velocity facilitates increased opportunity for binding interactions and chemokine signaling between leukocytes and endothelial cells. During the rolling phase, there is increasing integrin binding between endothelial cells and monocytes, which progressively slows the monocytes and leads to arrest and extravasation. In addition to this trafficking, monocyte binding to p-selectin results in increased sensitivity to chemokines in vitro. This cascade of adhesion and signaling interactions results in the activation, maturation, and trafficking of macrophages [103].

P-selectin has been shown to play a role in the recruitment of neutrophils and macrophages to the distal segments of nerves that have sustained partial sciatic nerve ligation [104]. P-selectin KO mice have diminished recruitment to the injury site and a reduction of inflammatory cytokines TNFα, IL-1β, and IL-6 [104]. This could be the result of downstream effects of inflammatory cytokines produced by cells populating the injury site, as it has been shown that IL-1 signaling results in increased expression of P-selectin by endothelial cells in mice. However, this effect has not been observed in humans.

BACE1 has been shown to cleave PSGL-1 in cell lines expressing endogenous or transfected BACE1, and no cleavage was observed in primary cells derived from BACE1 KO mice [102]. The increased macrophage recruitment in BACE1 KO mice could be the result of reduced cleavage of PSGL-1 on the surfaces of endothelial cells and monocytes, increasing the number of binding interactions to begin monocyte rolling and tethering (Fig. 4). Although, given that macrophage-specific deletion of BACE1 did not result in the increased levels of macrophage recruitment seen with global BACE1 KO [70], the role of PSGL-1 cleavage by BACE1 in macrophages seems to be largely irrelevant to the overall process.

## Conclusions

Regeneration following peripheral nerve injury is slow, but given the critical role macrophages play in regeneration, immunomodulation of these cells may present an opportunity to improve recovery and reduce morbidity associated with nerve injury. The substrate promiscuity of BACE1 could promote robust immunomodulation by acting on many pathways to alter the interactions between neurons, Schwann cells, and macrophages in the peripheral nerve microenvironment. Further study is necessary to determine the relative contributions of each cell type in the peripheral nerve microenvironment towards mediating the enhanced macrophage recruitment and activity phenotype observed in BACE1 KO mice. Previous experiments with macrophage-specific deletion of BACE1 did not result in enhanced recruitment of macrophages to the injured peripheral nerve [70], suggesting it would be worthwhile to pursue specific deletion of BACE1 in neurons, and particularly in Schwann cells. Given that Schwann cells express BACE1 and are critical for the mobilization of macrophages following nerve injury, they are a top candidate for further research. These experiments would help to determine if deletion of BACE1 in Schwann cells is sufficient to recapitulate the enhanced macrophage phenotype observed in global BACE1 KO mice and lend insight into the immunomodulatory role of BACE1 in the context of nerve injury. Additionally, these experiments would provide further evidence of the ways in which Schwann cells facilitate nerve regeneration through their signaling with macrophages.

## Data Availability

Not applicable.

## Abbreviations

ADAM: A disintegrin and metalloproteinase
APP: Amyloid precursor protein
BACE1: Beta-site APP cleaving enzyme 1, β-secretase
C3: Complement component 3
CCL2: C-C motif chemokine ligand 2
CNS: Central nervous system
DAMP: Damage associated molecular pattern
ECM: Extracellular matrix
Il-1α: Interleukin-1α
Il-1β: Interleukin-1β
Il-1R1: Il-1 receptor
Il-1R2: Interleukin 1 receptor 2
Il-1RA: Interleukin 1 receptor antagonist
Il-1RAcP: Interleukin 1 receptor accessory protein
Jag1: Jagged 1
KO: Knockout
LIF: Leukemia inhibitory factor
MMPs: Matrix metalloproteinases
NAD+: Nicotinamide adenine dinucleotide
NICD: Notch intracellular domain
NMN: Nicotinamide mononucleotide
NMNAT: Nicotinamide mononucleotide adenylyltransferase
NMJ: Neuromuscular junction
PAP-III: Pancreatitis-associated protein III
PKC: Protein kinase C
PMA: Phorbol myristate acetate
PNS: Peripheral nervous system
PSGL-1: P-selectin glycoprotein ligand-1
SARM1: Sterile alpha and TIR motif containing 1
SIRPα: Signal regulatory protein α
ST6Gal-1: ST6 beta-galactoside alpha-2,6-sialyltransferase 1
TNFα: Tumor necrosis factor α
TAM: Tyro3, Axl, Mer
VEGF: Vascular endothelial growth factor
WT: Wild type
